# A simple and easy *in vitro* model to test the efficacy of IV lines' needleless connectors against contamination

**DOI:** 10.1186/s40635-014-0027-9

**Published:** 2014-11-07

**Authors:** María Guembe, María Jesús Pérez-Granda, Luis Alcalá, Pablo Martín-Rabadán, Emilio Bouza

**Affiliations:** Department of Clinical Microbiology and Infectious Diseases, Hospital General Universitario Gregorio Marañón, C/ Dr. Esquerdo, 46, 28007 Madrid, Spain; Medicine Department, School of Medicine, Universidad Complutense de Madrid, Avda. Séneca, 2, 28040, Madrid, Spain; CIBER Enfermedades Respiratorias-CIBERES (CB06/06/0058), Madrid, Spain; Cardiac Surgery Postoperative Care Unit, Hospital General Universitario Gregorio Marañón, C/ Dr. Esquerdo, 46, 28007, Madrid, Spain

**Keywords:** Catheter colonization, Needleless connector, *In vitro* contamination, Manipulation model

## Abstract

**Background:**

Hub colonization after manipulation is responsible for 29% to 60% of catheter-related bloodstream infections (C-RBSI). Prevention can be achieved by the use of hub connectors, but its efficacy is generally based on instillation of high concentrations of microorganisms, which do not reflect the real contamination in daily practice. Our purpose was to create an *in vitro* model lasting long enough to be used for the comparison of the efficacy between various connectors against contamination simulating the real daily handling.

**Methods:**

The model consisted of 40 blood culture bottles with an inserted cannula with a needle-free closed connector. Twice a day, each line was manipulated while instilling 1 mL of two different fluids (saline and propofol). We manipulated the bottles as follows: ten bottles with clean gloves and disinfecting connectors with alcohol (controls), ten bottles with hands (no gloves), ten bottles with gloves impregnated with a 0.5 McFarland (MF) solution of *Staphylococcus aureus* (SA), and ten bottles with gloves impregnated with a 0.05 MF solution of SA. The bottles were incubated in a BACTEC System at 37°C under continuous agitation up to 10 days. When a bottle turned positive, 100 μL of the fluid was cultured and incubated followed by microorganism identification using standard procedures.

**Results:**

Overall, all bottles in the control group were negative at the end of the incubation time. In the three contamination experiments, almost all (38/40) bottles were positive during the incubation time. We only found differences regarding the median time to positivity (interquartile range (IQR)) between saline and propofol in the manipulation with SA 0.05 MF: 240 h (154.82 to 360.00) vs. 66 h (58.01 to 69.11), *p* = 0.008.

**Conclusions:**

A daily connector handling with 0.05 McFarland *S. aureus* solution while instilling saline proved to be a useful model lasting long enough to be used for the comparison of the efficacy of different types of closed needleless connectors against contamination.

**Electronic supplementary material:**

The online version of this article (doi:10.1186/s40635-014-0027-9) contains supplementary material, which is available to authorized users.

## Background

Intravascular catheters are widely used and are indispensable for proper patient management. However, its use implies several risks, which are mainly infectious. Catheter-related bloodstream infection (C-RBSI) is the more important entity which is associated to high rates of morbidity, mortality, and sanitary costs [1].

In general, in those catheters that are inserted for more than 8 days, infection is mainly acquired by an intra-luminal route (66%) because of hub contamination. Hub colonization after manipulation is responsible for 29% to 60% of catheter-related infections (CRI) [2].

Recently, it has been shown that the use of closed needleless connectors is effective against microorganism penetration by hub contamination. Some studies tested the *in vitro* efficacy of different needleless access devices to prevent the ingress of microorganisms [3-8]. However, these studies have heterogeneous designs, and they are all performed by instilling high concentrations of microorganisms through the catheter.

Our study purpose was to create an *in vitro* model lasting long enough be used to compare various connectors simulating the real daily handling of these devices based on blood culture bottles.

## Methods

### Setting

This *in vitro* study has being carried out in the Laboratory of the Clinical Microbiology and Infectious Disease Department of the Hospital General Universitario Gregorio Marañón.

### Study design

**Figure 1:**
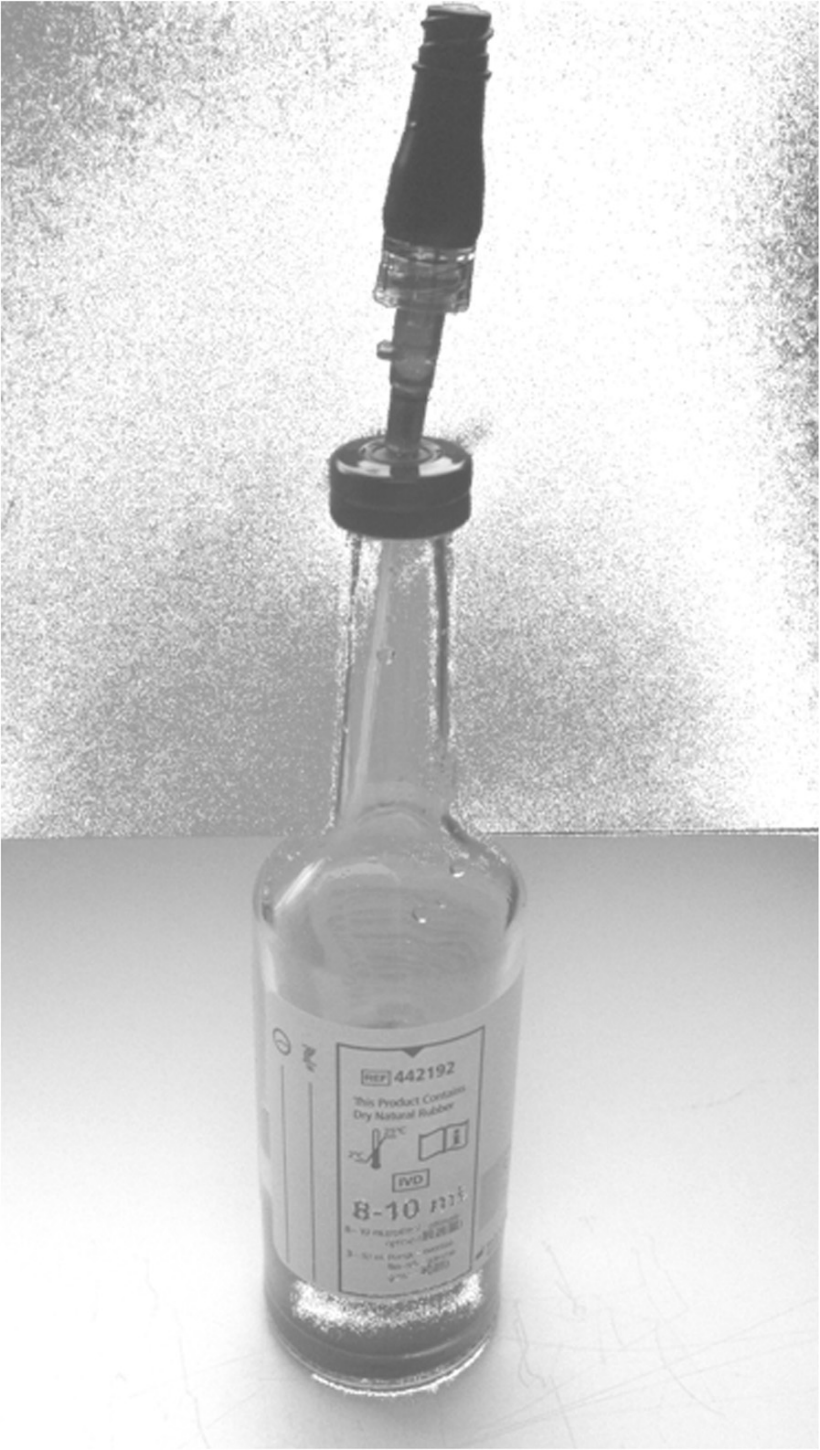
**Experimental model of the blood culture with the cannula and the CLAVE™.**

The model consisted of 40 blood culture bottles with a peripheral venous cannula inserted under sterile conditions and left in place (Figure 1). In each bottle, a disinfectable needle-free closed connector (CLAVE™, CareFusion, Spain) was used to close the cannulas. Twice a day, each cannula was manipulated while instilling 1 mL of either sterile saline or propofol. Manipulation of the bottles was divided into four models: ten bottles (five saline, five propofol) were manipulated with clean gloves and disinfecting the connector with alcohol (controls), ten bottles (five saline, five propofol) were manipulated without gloves (hands), ten bottles (five saline, five propofol) were manipulated with gloves impregnated with a 0.5 McFarland ATCC 29213 *Staphylococcus aureus* solution, and ten bottles (five saline, five propofol) were manipulated with gloves impregnated with a 0.05 McFarland ATCC 29213 *S. aureus* solution. Additional photograph files show this in more detail (see Additional files 1 and 2).

Before handling the connectors in the hand model, a base count and phenotypic identification of the colonizing microorganisms on the manipulators' hands were performed until the bottles became positive.

### Laboratory procedure

The bottles were incubated in a BACTEC 9120 System (Becton Dickinson Microbiology Systems, Maryland, DE, USA) up to 10 days at 37°C under continuous agitation. Each day, it was observed whether there was positivity in the bottle fluid (alert from the BACTEC 9120), and in case it occurred, 100 μL of the fluid was cultured into the blood, MacConkey, and Brucella agar and incubated under aerobic and anaerobic conditions for 48 h at 37°C. Microorganism identification was performed using standard procedures [9].

At the end of the incubation time (10 days, 20 mL), the negative bottles were also tested by culturing 100 μL of the fluid into the blood, MacConkey, and Brucella agar and incubated under aerobic and anaerobic conditions for 48 h at 37°C.

The different study variables were annotated in a data collection form.

### Statistical analysis

The qualitative variables appear with their frequency distribution*.* The quantitative variables are summarized as the median with interquartile range (IQR). Continuous variables were compared using the median test for non-normally distributed variables. The chi-square or Fisher exact test was used to compare categorical variables.

The Kaplan-Meier survival curve was used to compare the time to positivity with the degree of contamination.

All statistical tests were two-tailed. Statistical significance was set at *p* < 0.05 for all the tests. The statistical analysis was performed with SPSS 18.0.

### Ethics

This experimental design does not include human subjects and does not use human tissue or samples, so it was exempt from approval of the local ethics committee.

## Results and discussion

Overall, all bottles in the control group were negative at the end of the incubation time. In contrast, almost all bottles (38/40) in the three contamination experiments were positive during the incubation time. In the hand model with saline, we recovered the same *Staphylococcus epidermidis* strain in four out of the five bottles, which was phenotypically coincident with that isolated from the manipulators' hands. In the remaining bottle, we recovered a *Staphylococcus warneri* strain which was also the same strain we isolated from the manipulators' hands. In the hand model with propofol, all bottles recovered a *Micrococcus* sp., which was phenotypically coincident with that isolated from the manipulators' hands. In the remaining models with *S. aureus* with both saline and propofol, the ATCC 29213 *S. aureus* was recovered in 18 out of the 20 bottles (Table 1).

**Table 1:** **Characteristics of the experiment regarding contamination model and type of instillation fluid**

**Contamination degree**	**Instillation fluid**	**Colonizing microorganisms**	**MTP (IQR), h**	**Manipulator hand flora**
Hands^a^	Saline	*S. epidermidis*, *S. warnerii*	76.25 (65.92 to 125.01)	*S. epidermidis*, *S. warnerii,* CoNS, SV, *P. acnes*, *Micrococcus* sp.
	Propofol	*Micrococcus* sp.	74.91 (51.45 to 99.00)	*S. epidermidis*, *S. warnerii*, CoNS, SV, *P. acnes*, *Micrococcus* sp.
0.5 MF SA^b^	Saline	MSSA (ATCC 29213)	104.92 (45.97 to 239.94)	NA
	Propofol	MSSA (ATCC 29213)	147.89 (66.04 to 236.58)	NA
0.05 MF SA^c^	Saline	MSSA (ATCC 29213)	240.20 (154.82 to 360.00)	NA
	Propofol	MSSA (ATCC 29213)	66.11 (58.01 to 69.11)	NA
Control^d^	Saline	NA	NA	NA
	Propofol	NA	NA	NA

^a^Manipulation of the connector without gloves. ^b^Manipulation of the connector with gloves impregnated with a 0.5 McFarland solution of *Staphylococcus aureus* ATCC 29213. ^c^Manipulation of the connector with gloves impregnated with a 0.05 McFarland solution of *Staphylococcus aureus* ATCC 29213. ^d^Standard sterile manipulation with clean gloves and with disinfection of connectors using alcohol. MTP, median time to positivity; IQR, interquartile range; SA, *Staphylococcus aureus*; MF, McFarland; CoNS, coagulase-negative staphylococci.

**Figure 2:**
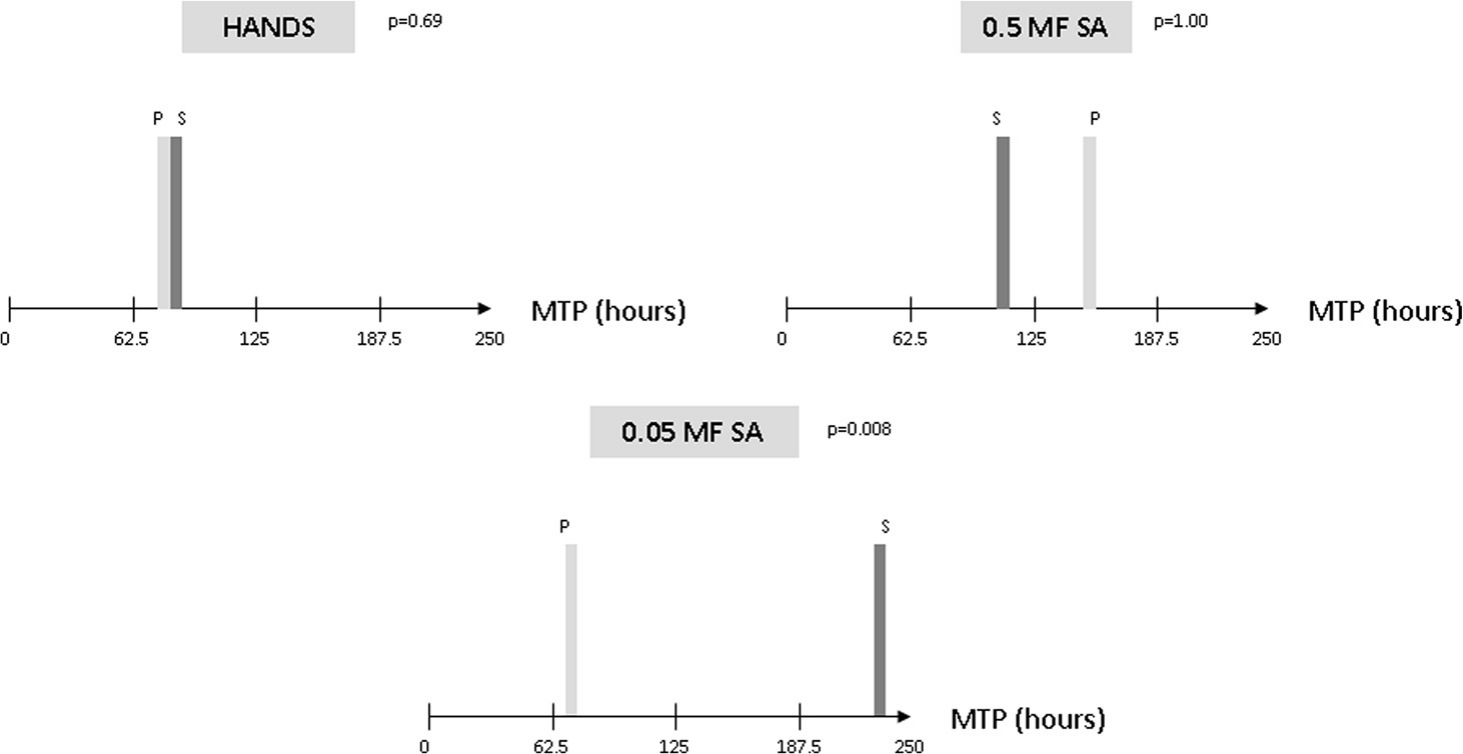
**Main results.** MF, McFarland; SA, *Staphylococcus aureus*; MTP, median time to positivity.

Regarding the median (IQR) time to positivity, there were no differences in either the hand model (saline 76 h (65.92 to 125.01) vs. propofol 75 h (51.45 to 99.00), *p* > 0.05) or in the 0.5 McFarland *S. aureus* model (saline 105 h (45.97 to 239.94) vs. propofol 148 h (66.04 to 236.58), *p* > 0.05). In contrast, in the 0.05 McFarland *S. aureus* model, the median (IQR) time to positivity was significantly higher when instilling saline than when instilling propofol: 240 h (154.82 to 360.00) vs. 66 h (58.01 to 69.11), *p* = 0.008. Moreover, when comparing the median (IQR) time to positivity between the three models with saline, the 0.05 McFarland *S. aureus* model was also significantly higher than the hands model: 240 h (154.82 to 360.00) vs. 76 h (65.95 to 125.00), *p* = 0.016 (Table 2, Figure 2).

**Table 2:** **Median time to positivity of the bottles comparing different contamination models and different fluids**

**Fluid**	**MTP (IQR), h**			***p***
	**Hands**	**0.5 MF SA**	**0.05 MF SA**	
Saline	76.25 (65.95 to 125.00)	104.92 (45.96 to 239.94)	240.20 (154.82 to 360.00)	*0.041* ^a^
Propofol	74.91 (51.44 to 98.99)	147.89 (66.03 to 236.58)	66.11 (58.01 to 69.11)	0.24
*p*	0.69	1.00	*0.008*	

^a^We found statistically significant differences between the 0.05 McFarland *S. aureus* model and the hand model. MTP, median time to positivity; SA, *Staphylococcus aureus*; MF, McFarland.

We have created an *in vitro* model of connector handling with gloves impregnated with a 0.05 McFarland *S. aureus* solution while instilling saline, lasting long enough to be used as a useful model to test the efficacy of closed needleless connectors.

Some recent *in vitro* studies used standard models of contamination to compare different needleless connectors against the capacity of microorganisms to ingress through the catheter lumen. However, some of them were performed using a two-phase method, in which, first, the connectors were contaminated with a single known microorganism (such as *S. aureus* or *S. epidermidis*, which is commonly associated to catheter infection) at different concentrations and, second, they were manipulated to instill contaminated infusion fluids [3,4,6,7,10,11]. But this methodology did not simulate the real practice of connector handling.

We compared two solutions of *S. aureus* of different concentrations by colonizing the surface of a blood culture bottle (while instilling sterile fluids), which simulates better the real daily practice, instead of instilling a contaminated fluid directly through the catheter. Besides, we tested not only a known concentration of a single known microorganism, but also the microorganisms from the flora of the manipulators' hands, as it has been demonstrated that these are highly colonized and could be a potential source of contamination [12]. With this model based on a simple daily handling, we have demonstrated that manipulation of connectors using gloves impregnated with a 0.05 McFarland solution of *S. aureus* while instilling sterile saline had longer times of positivity, allowing us to prove this model in the comparison of other types of connectors with time enough to detect differences between them regarding their efficacy to prevent the entry of microorganisms through the catheter.

Our study also supports the importance of disinfection before handling a connector, as in our model, all bottles manipulated without gloves turned positive up to 3 to 4 days after catheter insertion.

## Conclusions

A daily connector handling with a 0.05 McFarland *S. aureus* solution while instilling saline proved to be a useful model to test the efficacy of closed needleless connectors. Future studies must be performed with a larger sample and comparing other types of connectors.

## Additional files

Additional file 1:
**Manipulation with gloves.** The manipulation model showing the handling of the bottles while instilling the fluids using gloves impregnated with the *S. aureus* solution. Additional file 2:
**Manipulation without gloves (hands).** The manipulation model showing the handling of the bottles while instilling the fluids without gloves.

## Abbreviations

C-RBSI: catheter-related bloodstream infections
CRI: catheter-related infections
IQR: interquartile range

## References

[1] Kim JS, Holtom P, Vigen C (2011). Reduction of catheter-related bloodstream infections through the use of a central venous line bundle: epidemiologic and economic consequences. Am J Infect Control.

[2] Mermel LA, Allon M, Bouza E, Craven DE, Flynn P, O'Grady NP, Raad II, Rijnders BJ, Sherertz RJ, Warren DK (2009). Clinical practice guidelines for the diagnosis and management of intravascular catheter-related infection: 2009 update by the Infectious Diseases Society of America. Clin Infect Dis.

[3] Menyhay SZ, Maki DG (2008). Preventing central venous catheter-associated bloodstream infections: development of an antiseptic barrier cap for needleless connectors. Am J Infect Control.

[4] Yebenes JC, Delgado M, Sauca G, Serra-Prat M, Solsona M, Almirall J, Capdevila JA, Balanzó X (2008). Efficacy of three different valve systems of needle-free closed connectors in avoiding access of microorganisms to endovascular catheters after incorrect handling. Crit Care Med.

[5] Casey AL (2012) A laboratory comparison of microbial ingress into eight different needleless IV access devices, IDWeek. October 17–21. San Diego, CA, Poster No. 896

[6] Pulcini D (2012). Evaluation of fluid path colonization in needle-free connectors and biofilm formation in central venous catheters.

[7] Ryder M (2007). Transferencia De Bacterias A Través De Conectores Sin Aguja: Comparación De Nueve Dispositivos Diferentes.

[8] Ryder M (2012). Comparison of bacterial transfer and biofilm formation on intraluminal catheter surfaces among eight needleless connectors in a clinical simulated in vitro model.

[9] Baron EJ WM DW, Yagupsky P, Welch DF, Wilson DM (2005). Blood cultures IV.

[10] Menyhay SZ, Maki DG (2006). Disinfection of needleless catheter connectors and access ports with alcohol may not prevent microbial entry: the promise of a novel antiseptic-barrier cap. Infect Control Hosp Epidemiol.

[11] Yebenes JC, Martnez R, Serra-Prat M, Sauca G, Capdevila JA, Balanzo X, Palomar M (2003). Resistance to the migration of microorganisms of a needle-free disinfectable connector. Am J Infect Control.

[12] Pancholi P, Healy M, Bittner T, Webb R, Wu F, Aiello A, Larson E, Latta PD (2005). Molecular characterization of hand flora and environmental isolates in a community setting. J Clin Microbiol.

