# Supplementary figures and images for: A simple and easy in vitro model to test the efficacy of IV lines' needleless connectors against contamination
Source: Intensive Care Med Exp. 2014 Nov 7;2:27. doi: 10.1186/s40635-014-0027-9 (PMC4513010; doi:10.1186/s40635-014-0027-9)

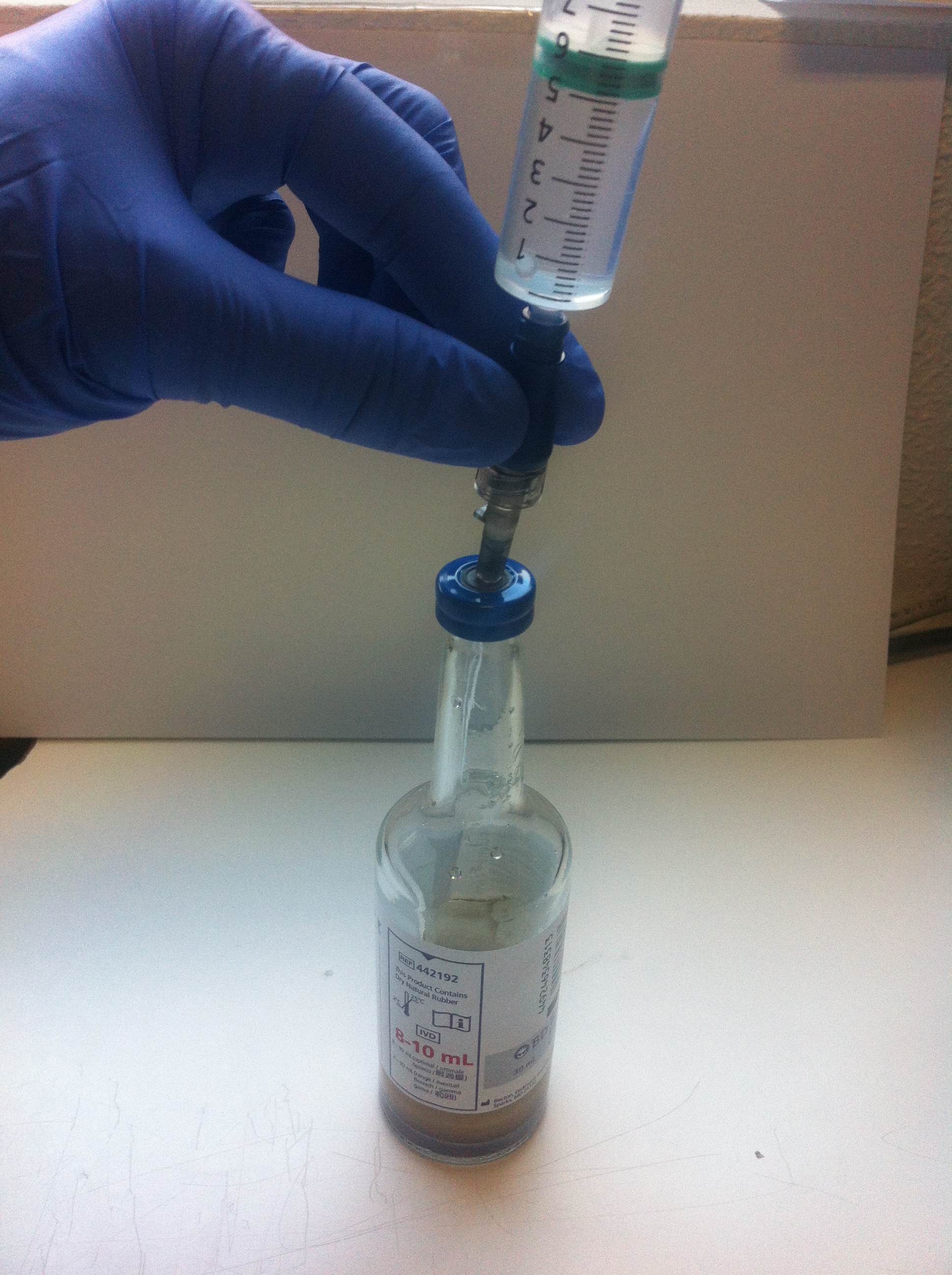

Supplement: Additional file 1: — Manipulation with gloves. The manipulation model showing the handling of the bottles while instilling the fluids using gloves impregnated with the S. aureus solution. [file 40635_2014_27_MOESM1_ESM.jpeg]

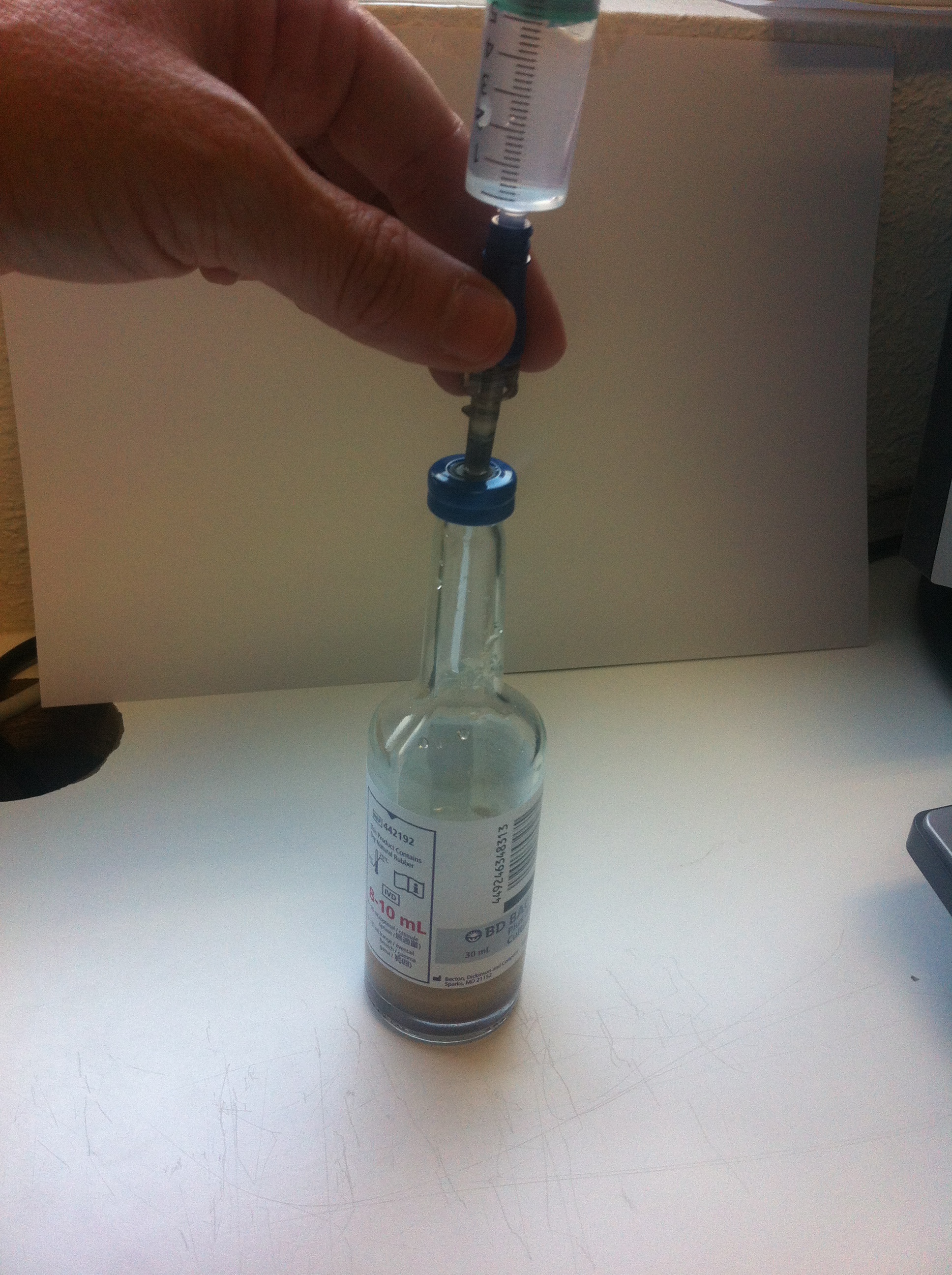

Supplement: Additional file 2: — Manipulation without gloves (hands). The manipulation model showing the handling of the bottles while instilling the fluids without gloves. [file 40635_2014_27_MOESM2_ESM.jpeg]
